# Prospective register-based study of the impact of immigration on educational inequalities in mortality in Norway

**DOI:** 10.1186/s12889-015-1717-2

**Published:** 2015-04-11

**Authors:** Jon Ivar Elstad, Einar Øverbye, Espen Dahl

**Affiliations:** NOVA, Oslo and Akershus University College, Oslo, Norway; Faculty of Social Sciences, Oslo and Akershus University College, Oslo, Norway

**Keywords:** Healthy migrant effect, Migration, Mortality, Socioeconomic inequality, Trends

## Abstract

**Background:**

Differences in mortality with regard to socioeconomic status have widened in recent decades in many European countries, including Norway. A rapid upsurge of immigration to Norway has occurred since the 1990s. The article investigates the impact of immigration on educational mortality differences among adults in Norway.

**Methods:**

Two linked register-based data sets are analyzed; the first consists of all registered inhabitants aged 20–69 in Norway January 1, 1993 (2.6 millions), and the second of all registered inhabitants aged 20–69 as of January 1, 2008 (2.8 millions). Deaths 1993–1996 and 2008–2011, respectively, immigrant status, and other background information are available in the data. Mortality is examined by Cox regression analyses and by estimations of age-adjusted deaths per 100,000 personyears.

**Results:**

Both relative and absolute educational inequality in mortality increased from the 1993–1996 period to 2008–2011, but overall mortality levels went down during these years. Immigrants in general, and almost all the analyzed immigrant subcategories, had lower mortality than the native majority. This was due to comparatively low mortality among lower educated immigrants, while mortality among higher educated immigrants was similar to the mortality level of highly educated natives.

**Conclusions:**

The widening of educational inequality in mortality during the 1990s and 2000s in Norway was not due to immigration. Immigration rather contributed to slightly lower overall mortality in the population and a less steep educational gradient in mortality.

## Background

In many European countries, socioeconomic differences in mortality have widened [1-3]. This is also the case in Norway [4,5]. The persisting and partly increasing socioeconomic differences in mortality, also in advanced welfare states with relatively small income inequalities, have puzzled researchers. Several explanations have been put forward [6,7], but immigration is seldom taken into consideration when the issue is examined. In this study, we ask how mortality inequalities in Norway have been influenced by immigration. Immigrants will often belong to lower socioeconomic strata [8]. If immigrants who enter these strata have higher mortality than the natives in these strata, wider socioeconomic differences in mortality may follow. If, on the other hand, immigrants in lower strata are healthier than their native counterparts, immigration may narrow the size of socioeconomic inequalities in mortality. Through similar mechanisms the mortality of high-status immigrants may influence the pattern of socioeconomic inequalities in mortality in the total population.

How immigration impacts on socioeconomic inequalities in mortality is partly an effect of the overall health situation of immigrants. Compared to the mortality level in the native non-immigrant majority, lower mortality among immigrants – also immigrants from non-Western countries – has been observed in many countries, such as Norway [9], Denmark [10], Sweden [11], The Netherlands [12,13], Germany [14], France [15], Australia [16], and U.S.A. [17]. This is often interpreted as a “healthy migrant effect” [18,19]. Usually, this term refers to health-selective processes in the immigrants’ country of origin. As migration requires resources not only in means and motivation, but also in health, those who migrate will regularly have lower mortality risk than those who stay. However, this type of health-selective process does not necessarily imply that immigrants have better health than the native population in the destination country [20]. There are also other reasons for relatively good health among immigrants. Lower immigrant mortality could be due to healthier lifestyles, for instance, less use of alcohol or less smoking, than among the natives [21]. Screening by immigration authorities could also play a role. Some countries, e.g., U.S.A., Canada and Australia, limit access on the basis of the immigrant’s likely economic success, assessed by a skill-based points system. One effect of this could be that immigrants will be relatively healthy [22].

Research has, on the other hand, also discovered many instances of relatively poor health among immigrants, compared to the native majority. This appears, for instance, to be the case in the U.K. for Irish [23] and several other immigrant categories [24]. Nordic immigrants to Sweden, Finns in particular, seem to have relatively high mortality [25]. Mortality among men with Turkish origin living in The Netherlands seems to be high [13]. For specific causes of death, immigrant mortality rates are often higher than the native level [10,13,18], and high levels of illness and morbidity in some immigrant categories have been demonstrated [26,27].

Comparatively high levels of ill health among immigrants could be due to, for instance, a precarious childhood in the country of origin, strain linked to the migration process itself, and poverty, discrimination, occupational hazards, minority stress, and other detrimental exposures in the receiving country [20,28]. Health may also vary with the reason for migration. Work immigrants will often be relatively healthy, while the health of refugees and asylum seekers could be influenced by traumatic exposures [22]. A Danish study found that although refugees had lower mortality than the native population, they had higher mortality than family-reunited immigrants [10]. It has furthermore been suggested that “large” welfare states will attract immigrants with health problems, since access to health care could be easier [29]. Moreover, an initial health advantage may fade away over time [17,18,20,23]. The health status of immigrants may approach that of their native-born counterparts if immigrants adopt prevailing lifestyles and experience life conditions similar to the native population. As they often are confronted with more unfavourable material standards of living than the natives, and are not seldom exposed to unhealthy work environments, discrimination, insufficient health care, and other adverse circumstances, immigrants may even experience a particularly rapid health deterioration with increasing age [30].

Accordingly, the impact of immigration on socioeconomic inequalities in mortality will depend on many circumstances – not the least on the pattern of socioeconomic inequalities in health *within* the immigrant population. Some studies indicate that the socioeconomic gradient in mortality among immigrants and ethnic minorities is comparatively flat; sometimes called the immigrant health paradox [12,30,31]. However, the size of socioeconomic mortality differences seems to vary considerably between immigrant categories [32,33], but many countries lack studies of socioeconomic inequality in mortality in their immigrant population.

In this paper, the aim is to examine the role of immigration for the educational inequalities in mortality in Norway. Since the 1980s, the Norwegian economy has been steadily growing and fairly unaffected by the economic downturn in Europe in the early 1990s and the international financial crisis after 2008 [34]. Household incomes have increased in real terms, poverty rates are low, and the welfare state has not contracted. Income inequalities have increased somewhat, but Norway is still among the most egalitarian countries in Europe [35].

Given these social and economic conditions, public health theories would suggest that the magnitude of health inequalities would stabilize or perhaps even be reduced [6,7]. Nevertheless, mortality studies [4,5,36,37] indicate increasing mortality inequalities for several decades, both in relative and absolute terms; it should be noted, however, that the most recent study suggests that absolute inequalities among men has not increased since 2000 [4]. At the same time, the immigrant population in Norway has grown considerably. In 1990, 4.0% of all registered inhabitants were immigrants (defined as inhabitants with foreign-born parents); in 2008, this percentage had more than doubled to 9.7% [38]. In 1990, 1.8% of the population in Norway had a country background from Asia, Africa, Turkey, and Latin America, increasing to 5.2% in 2008. Thus, unlike countries with a long history of immigration (e.g., The Netherlands, U.K., U.S.A.), Norway has had a rapid surge in recent years, both work immigrants (e.g., East Europeans after 2004), family reunions (e.g., Pakistani), and refugees and asylum seekers (e.g., from the former Yugoslavia, Iraq, Sri Lanka, Somalia, etc.).

On this background, the aim of the present study is to analyze the role of immigration for the development of educational differences in mortality in Norway. We examine the relationship between education and all-cause mortality and how it has changed since the mid-1990s, and we compare natives’ and immigrants’ death rates, overall and in different educational categories, in order to examine the impact of immigrants’ mortality on the mortality differences in the total population.

## Methods

### Data

The study is based on two separate, linked register databases, prepared by Statistics Norway. The first data set consists of all inhabitants in Norway 1^st^ of January 1993 (i.e., listed in the population register as permanently residing in Norway, both Norwegian and foreign citizens). The second data set consists, correspondingly, of all inhabitants 1^st^ of January 2008. The data have individual information from several public registers, linked by the unique personal identification number. For most variables, missing values are few and negligible – an important exception is the educational variable, however, which will be discussed below. Information about deaths 1993–1996 was available in the 1993 data; similarly, the 2008 data have mortality information 2008–2011.

The data are well suited for analyses of mortality inequalities, but, in addition to missing educational information, there are some other weaknesses. Information on country or world region origin is only given in 2008, but not in 1993, and reasons for immigration (e.g., work, studies, family re-union, refugee, asylum seeker) are not available in these data. Moreover, undocumented immigrants are lacking in the registers. This is not likely to affect the main results, however, as undocumented immigrants are not many; their number were estimated to 18,000 in 2006 [39] at a time when there were about 400,000 registered immigrants [38].

The analyzed samples consist of inhabitants aged 20–69 years at baseline (born 1923–1972 in the 1993 data; 1938–1987 in the 2008 data). The age restriction was made because there are few immigrants from non-Western countries aged 70+. The immigrant status classification is based on information about country of birth for the individual and his/her parents. In the present study, we compare *natives* (i.e., born in Norway and neither parent born outside Norway) with *immigrants*, defined here as individuals with permanent residency in Norway who were born outside Norway of parents with no Norwegian connection. In order to highlight the contrast between natives and immigrants, those with a “mixed” background (e.g., born in Norway by two immigrant parents, or having one immigrant and one native parent) are excluded from the analyses (3.2% of the total 20–69 age population in 1993, 4.7% in 2008).

### Variables

Mortality information is available in terms of month of registered death during the follow-up years 1993–1996 and 2008–2011. The estimation of exposure time, i.e., personyears, is straightforward for those with reported month of death, or when the data clearly indicate that the individual was alive and residing in Norway at the end of 1996 or 2011, respectively. In the 2008 file, information on emigration 2008–2011 reported to the population register has furthermore been used for calculating personyears. In the 1993 data, information about emigration 1993–1996 was absent. To handle this, individuals in the 1993 data were classified as emigrated 1993–1996 if death was not registered during these years, but neither was there any information about municipality of residence in 1996. These assumed emigrated individuals were assigned two personyears, i.e., supposed to have emigrated, on average, in the midst of the 1993–1996 period.

Age information was only given in 10-years bands (age 20–29, age 30–39, etc.) because of anonymity precautions required by the Norwegian Data Protection Authority.

Educational information was obtained from Statistic Norway’s register on “highest” education, coded by the ISCED scheme [40]. Education was classified into three levels: Lower education (primary or less education plus lower levels of secondary education); medium education (higher levels of secondary education); and higher education (comprising college and university levels). Mandatory reporting to Statistics Norway from all approved Norwegian educational institutions ensures high-quality information for those who have completed their education in Norway. Thus, very few natives lacked educational information (1.3% in the 1993 data, 0.7% in the 2008 data). Among the immigrants, however, information about education lacked for as many as 37.8% in the 1993 data and for 19.9% in the 2008 data. Those immigrants who completed their education in Norway will automatically be registered, and educational information is also routinely collected for refugees and asylum seekers, and for immigrants who work in occupations where documented educational qualifications are required, such as physicians and nurses. For other immigrants, educational information depends mostly on voluntary self reports. In order to collect educational data for immigrants without a record in the educational register, Statistics Norway carried out surveys in 1991, 1999, and 2011 [8]. These surveys have improved the information about immigrants’ education, but as non-response has been considerable (in 2011, the response rate was 64%), educational information was still lacking for about a fifth of the immigrants in the 2008 data.

### Statistical analyses

We analyze both *relative* inequalities in mortality, in terms of hazard ratios obtained from Cox regression analyses, and *absolute* levels of mortality and *absolute* inequalities in mortality, using age-adjusted, all-cause, number of deaths 1993–1996 and 2008–2011 per 100,000 personyears. Analyses are made separately for men and women, with IBM SPSS Version 21. The direct age standardization has taken the proportional age distribution in the native majority in 2008 as the standard: Age 20–29 = 0.184; 30–39 = 0.218; 40–49 = 0.223; 50–59 = 0.209; and 60–69 = 0.166. This age distribution is close to the distribution in the new European Standard Population [41].

The lack of educational information for a considerable part of the immigrants is a challenge for this study, although this drawback will not affect the estimation of overall immigrant mortality. Missing educational information complicates the estimation of educational inequalities in mortality both in the immigrant population and in the total population, however. We have handled this difficulty by, first, analyzing only those who actually had educational information, and second, by pooling those without educational information with lower education. Both approaches have drawbacks. As regards the first, many immigrants fall out of the analyses; but the second approach, i.e., pooling those without educational information together with the low educated, will be erroneous to some extent since these individuals will certainly be distributed across the entire educational hierarchy. Nevertheless, the latter approach is not entirely arbitrary since it is likely that many of the immigrants who lacked educational information, had relatively short education. In the 2008 data, for instance, separate analyses (not shown) indicate that a large proportion of those without educational information were recent immigrants from Poland or other new EU member countries in East Europe, i.e., having a country background typical for work immigrants who mostly enter manual or low-skilled occupations in Norway.

In the last part of the analyses, the educational gradient among immigrants according to country or world region origin is examined. This was only possible in the 2008 data, since no information on immigrants’ country background was available in 1993. Mortality rates 2008–2011 and educational inequality in mortality among natives and in six immigrant categories are displayed. Because of few deaths in some categories, only a two-level educational classification was feasible in this analysis.

### Ethics

Data were provided by Statistics Norway for public health research projects funded by the Research Council of Norway (grant numbers 163970 and 221000). The projects have been approved by the Norwegian Data Protection Authority. The data files were constructed by linking information from public registers. Before making data available for research, Statistics Norway removed the personal identification number and other information which potentially could be used for identification of individuals.

## Results

Baseline number of analyzed persons aged 20–69 were about 2.6 millions in the 1993 data and 2.8 millions in the 2008 data (Table 1). The percentage immigrants was 5.2% in the 1993 sample and 10.5% in the 2008 sample. Among the 2008 immigrants, 25.6% had a background from Western Europe, 22.8% from Central/Eastern Europe, 45.3% from Africa and Asia, and the remaining 6.3% from America and Oceania. In both data files, a smaller percentage among the immigrants (about 8%) than among the natives (about 15%) were aged 60–69. The proportion having high education was fairly similar for natives and immigrants in both samples. The proportion with lower education was lower among immigrants than among natives, however, especially in the 1993 data when many immigrants (38%) lacked educational information. This suggests that many immigrants without educational information had actually relatively little schooling.

**Table 1:** **Sample description: total number, number of personyears and distribution by gender, age and education among natives and immigrants**

	**1st January 1993 sample, age 20-69**			**1st January 2008 sample, age 20-69**		
	**All**	**Natives**	**Immigrants**	**All**	**Natives**	**Immigrants**
Baseline study cohort	2,600,945	2,465,694	135,251	2,840,119	2,541,288	298,831
Row percent	100.0	94.8	5.2	100.0	89.5	10.5
Column percentages						
Women %	49.5	49.6	47.2	49.3	49.3	48.9
Age 20–29%	24.4	24.3	26.4	18.5	17.9	23.7
Age 30–39%	23.3	22.9	31.3	22.4	21.5	30.2
Age 40–49%	22.6	22.6	22.2	22.5	22.2	24.5
Age 50–59%	15.0	15.1	12.1	20.5	21.2	14.0
Age 60–69%	14.7	15.1	8.0	16.1	17.1	7.5
Education						
Missing %	3.2	1.3	37.8	2.7	0.7	19.9
Lower %	48.5	49.8	24.5	36.6	37.3	30.9
Medium %	27.7	28.3	16.9	30.0	31.1	20.6
Higher %	20.6	20.5	20.9	30.7	30.9	28.6
No. deaths	46,352	44,867	1,485	39,130	36,844	2,286
Personyears	10,244,727	9,743,461	501,266	11,197,343	10,072,958	1,124,385

Natives = no immigrant connection; immigrants = foreign-born with foreign-born parents; others (e.g., second-generation immigrants, mixed immigrant/parents, etc. 3.2% of total 20–69 population in 1993, 4.7% in 2008) are not included in the analyses. No. (number) deaths/personyears refer to 1993–1996 and 2008–2011 respectively.

Table 2 indicates that for the total population aged 20–69, *relative* educational inequalities in mortality increased from 1993–1996 to 2008–2011. For men *with* educational information, the hazard ratios (HRs) for lower education (reference higher education) were 1.89 in the 1993 sample and 2.32 in the 2008 sample; the rise in HRs when missing educational information was pooled with lower education was from 1.90 to 3.13. The corresponding HRs for women (natives and immigrants together) did also increase, from 1.62 to 2.05 (with educational information) and from 1.64 to 2.72 (missing and lower education pooled).

**Table 2:** **Relative educational inequalities in mortality, age 20–69 at baseline, age adjusted, Cox regression, hazard ratios (HR) with 95% CI**

	**1st January 1993 sample, age 20-69**			**1st January 2008 sample, age 20-69**		
	**All**	**Natives**	**Immigrants**	**All**	**Natives**	**Immigrants**
**Men**						
Education						
Higher	1.0	1.0	1.0	1.0	1.0	1.0
Medium	1.34	1.34	1.42	1.45	1.46	1.30
95% CI	1.29-1.40	1.28-1.40	1.13-1.78	1.39-1.52	1.39-1.53	1.09-1.56
Lower	1.89	1.90	1.66	2.32	2.37	1.61
95% CI	1.82-1.96	1.83-1.97	1.35-2.04	2.23-2.41	2.27-2.47	1.38-1.88
Lower incl. missing	1.90	1.92	1.50	3.13	3.19	2.67
95% CI	1.83-1.97	1.85-1.99	1.24-1.82	3.01-3.25	3.06-3.32	2.33-3.07
**Women**						
Education						
Higher	1.0	1.0	1.0	1.0	1.0	1.0
Medium	1.18	1.17	1.40	1.24	1.25	1.11
95% CI	1.10-1.28	1.08-1.27	1.04-1.88	1.16-1.33	1.17-1.34	0.86-1.43
Lower	1.62	1.62	1.70	2.05	2.09	1.45
95% CI	1.53-1.71	1.53-1.72	1.35-2.14	1.95-2.16	1.99-2.21	1.20-1.76
Lower incl. missing	1.64	1.64	1.57	2.72	2.77	2.34
95% CI	1.55-1.73	1.55-1.74	1.26-1.95	2.59-2.86	2.63-2.92	1.97-2.79

Lower incl.missing: Missing educational information pooled with lower education. Note: HRs for medium education will be practically identical, no matter whether missing education is included in, or excluded from, the lower education category; here, HRs for medium education is estimated in samples where those without educational information have been excluded.

The Cox regression analyses performed separately for natives and immigrants do not suggest that immigrants’ mortality pattern could explain the overall increase in relative educational inequalities. HRs increased from the 1993 to the 2008 sample in much the same way (or slightly more) for natives analyzed separately, as for the entire population. The HRs for lower education, both when excluding and when including missing educational information in the lower education category, were usually smaller among immigrants than natives. In the 2008 sample for men, for example, HRs for lower education including missing were 2.67 for immigrants and 3.19 for natives (the 95% CI intervals touch each other, however). An exception is the female part of the 1993 sample, where immigrants and natives had similar HRs.

However, comparing HRs across different samples is problematic [42], for instance because the absolute mortality level in the reference category may differ between samples. Therefore, analyses of *absolute* mortality rates may illuminate the topic of this paper better. Table 3 demonstrates that overall mortality declined markedly between the two periods, from 693 deaths per 100,000 personyears to 444 among men, and from 365 to 273 among women. Furthermore, overall mortality was clearly *lower* for immigrants than natives (exception: women, 1993 sample); thus, for males, mortality rates were 625 (immigrants) vs. 697 (natives) in the first period, and 402 vs. 448, respectively, in the second period.

**Table 3:** **Absolute educational inequalities in mortality (deaths per 100,00 personyears, age-adjusted), 1993-1996 and 2008-2011**

	**1st January 1993 sample, age 20-69**			**1st January 2008 sample, age 20-69**		
	**All**	**Natives**	**Immigrants**	**All**	**Natives**	**Immigrants**
**Men**						
Total	693	697	625	444	448	402
Education						
Higher	445	445	477	214	214	228
Medium	599	598	648	317	319	300
Lower	823	825	743	500	514	356
Lower incl. missing	824	833	667	672	691	576
Difference*	379	388	190	458	477	348
**Women**						
Total	365	365	357	273	276	222
Education						
Higher	243	243	243	140	141	136
Medium	300	296	356	174	177	148
Lower	393	392	425	285	293	195
Lower incl. missing	396	397	395	374	385	307
Difference*	153	154	152	234	244	171

Lower incl.missing: Missing educational information pooled with lower education. Difference* = lower incl. missing minus higher.

Table 3 indicates furthermore that, when measured as the difference between the lower (including missing) and higher educational category, absolute educational inequality in mortality *increased* in the total population from 1993–1996 to 2008–2011. For men, the increase was from 379 to 458, for women from 153 to 234.

The increase in educational inequality can be decomposed into contributions from natives and from immigrants. Absolute inequalities were slightly larger for natives analyzed separately than for the entire population (exception: women, 1993 sample). The increase in inequality from 1993 to 2008 was larger among natives than in the entire population. Compared to the mortality of higher educated natives, the mortality of higher educated immigrants was similar for women and slightly higher for men, in both periods. Mortality levels among lower educated immigrants, on the other hand, were *markedly* lower than mortality among lower educated natives, in both periods (exception: women in the 1993 sample). The overall effect of these patterns has obviously been that immigrants’ mortality had an overall *moderating* influence on the size of absolute educational inequalities. The educational gradient in each of the two periods was constrained by the mortality patterns among immigrants, as was the widening of inequality from the first to the second period.

In the 2008 sample, immigrants could also be classified according to country or world region origin. Table 4 shows that for *men*, when compared with natives’ mortality rates, overall mortality was clearly lower for immigrants from Africa, Turkey/Iraq/Iran, and “other Asia”, but similar to the natives’ level when coming from East Europe, Pakistan, and Western countries. For Pakistani *women*, overall mortality was higher than for native women, but the other female immigrant categories had lower overall mortality than native women. Even when a crude two-level educational classification was used, the number of deaths for those with higher education were too small for meaningful calculations of mortality rates in several world region categories. Nonetheless, Table 4 suggests that the mortality difference between lower/missing and higher/medium education among Western immigrants of both genders, and among East European men, was similar to the educational gradient among the natives. In male immigrants from Africa and Asia, educational inequality in mortality appeared as much narrower than in the native population.

**Table 4:** **Absolute educational inequalities in mortality, 2008-2011, by country or world region origin**

	**Number of**		**Deaths per 100,000 personyears, age-adjusted**			
				**Educational level**		
	**Persons at baseline**	**Deaths**	**All**	**High, medium**	**Lower, missing**	**Diff.**
Men						
Natives	1,287,949	22,731	448	267	691	424
Western country	44,374	643	428	254	693	439
East Europe	36,818	285	446	285	711	426
Africa	17,745	118	319	195	414	219
Turkey/Iraq/Iran	18,040	124	352	258	438	180
Pakistan	7,295	98	441	(−)	543	(−)
Other Asia	23,435	176	315	220	410	190
Women						
Natives	1,253,339	14,113	276	154	385	231
Western country	38,946	327	219	144	358	214
East Europe	31,337	135	179	111	280	169
Africa	14,112	62	253	(−)	283	(−)
Turkey/Iraq/Iran	13,416	43	226	(−)	280	(−)
Pakistan	6,765	58	349	(−)	394	(−)
Other Asia	34,653	142	214	176	237	61

(−) = less than 30 deaths. Western country = North and West Europe, North America, Australia and New Zealand. East Europe = mostly from Poland, former Soviet Union, and former Yugoslavia. Diff. = Lower/missing minus High/medium.

## Discussion

Mortality in the total population of Norway for the analyzed age categories (20–69 at baseline) was considerably reduced from the 1993–1996 to the 2008–2011 period. In both periods, clear mortality differences between the natives and the immigrants could be observed. Overall age-adjusted mortality in the immigrant male population was around 10 per cent lower than overall mortality among native men in both periods, and the mortality of female immigrants in 2008–2011 (but not in 1993–1996) was markedly lower than the overall mortality among native women.

Moreover, educational inequalities in mortality, both in relative and absolute terms, increased in the total population between the two time periods. This development could not be explained by immigration, however. Overall mortality was generally lower among immigrants than among natives. Both relative and absolute educational differences in mortality among men were smaller in the immigrant population than among natives in both periods, and the same pattern existed for women in the 2008–2011 period. The mortality advantage of immigrants, compared to natives, did not arise because of lower mortality among high-educated immigrants, but because mortality rates in lower educated categories were clearly lower among immigrants than among lower educated natives.

Taken together, these patterns imply that the widening of educational inequality in mortality in the total resident population in Norway has not been due to immigration. On the contrary, the general impact of immigration has been to diminish the magnitude of educational inequalities in mortality, both in the 1993–1996 and the 2008–2011 period, and to inhibit the increase in educational inequalities between these two periods.

One might comment that this is hardly a surprising result. The immigrants’ share of the population aged 20–69 is not very large (even though it doubled during the observation period from 5% to 10%). One could argue that this implies that immigrants’ mortality could not influence the overall education-mortality relationship to any large extent. Nonetheless, if there are particular mortality patterns in one tenth of the population, the overall educational inequalities in mortality could be influenced, and empirical enquiry was therefore required in order to determine the role of immigration on the educational gradient in mortality in Norway.

The results could be interpreted as demonstrating the “healthy migrant effect” in Norway. It is likely that those who have migrated to Norway will be more healthy on average than the remaining local population. An interesting tendency, also found in previous studies [12], is moreover that educational inequalities in mortality seemed less marked among immigrants (especially non-Western immigrants) than in the native population. This pattern was primarily due to low mortality among low educated immigrants, compared to natives with low education. This could be a specific “healthy migrant effect” in the sense that for those who lack educational resources, health resources will be particularly important for being able to emigrate.

However, immigrants’ health situation is likely to be influenced by a wider set of circumstances than the health-selective processes involved in migration [18-20]. The “healthy migrant effect”, as commonly understood, offers an explanation for health differences between migrants and non-migrants in the country of origin, but it does not follow from this that health among immigrants will be better than health among the natives in the receiving country. A broad set of factors, linked both to pre-migration background, the migration process itself, and the exposures in the “new” country, is likely to be involved. Moreover, immigrants are not a homogeneous category, but differ according to where they come from, why they have migrated, and which positions they attain in the host country [19]. Such variations are indicated in this study by the mortality differences between immigrants from different world regions. One further issue for research is how immigrants’ health, relative to natives’, differs according to types of diseases and causes of death [18]. Still another challenge is to apply a life course perspective on immigrant health and examine how duration of stay in the host country influences how health develops [18,20]. Thus, many types of factors combine in generating a particular profile of health among immigrants at a specific time point. Whether the immigrant-native mortality differences disclosed in this study will persist, is difficult to predict – the answer to this question requires empirical investigations.

A particular health-selective process which could influence immigrants’ mortality level is the so-called “salmon effect”: when growing old or sick, immigrants may re-emigrate to their native country and perhaps die there without notifying the population register in their “new” country. Mortality estimates, especially as regards non-Western immigrants, could be unduly favourable because of re-emigration [20]. However, a recent study suggests that re-emigration is a rare event among seriously ill immigrants in Denmark [43], probably because access to health care is good. As provision of health care in Norway resembles the Danish situation, it is not likely that a “salmon effect” has seriously biased the findings in the present study.

A strong aspect of the present study is the data sources. Norwegian public registers have a reputation for high quality and good updating routines. Errors, for instance as to information about age, country of birth, or death, are likely to be few, and by analyzing the entire registered population, errors due to sample bias will be minimized. However, undocumented immigrants have not been considered in this study, but when analyzing whether the increase in educational differences in mortality in the *registered* population can be explained by immigration, it is not mandatory to include undocumented immigrants, i.e., the unregistered population, in the study.

Nevertheless, the substantial proportion of immigrants lacking educational information, in particular in the 1993 data, makes some results uncertain. This may even be aggravated by difficulties in “translating” educational information among immigrants into the Norwegian educational hierarchy. Improved collection of education data among immigrants is necessary to minimize this source of error.

When considering the results, one should also take into account that the immigrant population in Norway, especially those from parts of Africa, the Middle East, and Asia, is rather young, and the number of deaths in several categories are often small (as indicated by Table 4). This is also illustrated by the wide confidence intervals around the hazard ratios for immigrants reported in Table 2, but under-communicated in Table 3 since no confidence intervals are given there. The fact that the immigrant population is young and have a limited number of deaths implies that other health indicators are needed for exploring the immigrant health situation better.

In the present study, education was used to indicate socioeconomic position, in line with many previous studies on mortality differences [1,4]. Also other indicators of socioeconomic position, such as occupation, employment status, or income, could have functioned in similar ways. Although the frequency of missing educational information is problematic in this study, we chose to rely on the education indicator since it is less influenced by an individual’s current health, and the missing information problem would also be significant if other indicators were employed. It should be noted, however, that the present study does not presuppose any causal relation between education and mortality. Education is used here only in order to describe patterns of social inequalities, while analyses of the causal processes which generate mortality risk are beyond the scope of this study.

This study was motivated by the hypothesis that immigration could have contributed to the persisting and partly increasing socioeconomic inequalities in mortality. The results negate this hypothesis, at least in the Norwegian context, and other approaches are clearly required in order to resolve this issue. As pointed out in the introduction, educational differences in mortality have widened in many European countries [1-3], and this has coincided with increases in the immigrant populations. This suggests that a topic for further research could be to examine the topic adressed by this paper in other European countries.

## Conclusion

In recent decades, immigration to Norway has increased. By the late 2000s, about 10% of the adult population were immigrants, many of them from East Europe, Africa and Asia. In the same decades, both relative and absolute educational inequalities in mortality in the population have increased. The aim of this study was to examine whether immigration has been a factor in the development of mortality inequalities. The results of the analyses indicate the contrary. Due to lower mortality rates in the immigrant population as a whole, and in particular due to low mortality among non-Western low educated immigrants, compared to low educated natives, the role of immigration has been to moderate the educational gradient in mortality and to constrain the widening of educational inequalities in mortality.
